# ACCURACY OF ULTRASOUND FOR SARCOPENIA ASSESSMENT IN DIGESTIVE TRACT CANCER PATIENTS

**DOI:** 10.1590/S0004-2803.24612025-127

**Published:** 2026-05-25

**Authors:** Tassiane de Paula SUDBRACK, Katia BARÃO, Nora Manoukian FORONES

**Affiliations:** 1 Universidade Federal de São Paulo, Escola Paulista de Medicina, Departamento de Medicina, Programa de Pós-Graduação em Gastroenterologia, São Paulo, SP, Brasil.; 2 Universidade Federal de São Paulo, Escola Paulista de Medicina, Departamento de Medicina, Disciplina de Gastroenterologia, São Paulo, SP, Brasil.; 3 Universidade Federal de São Paulo, Escola Paulista de Medicina, Departamento de Medicina, Setor de Oncologia, Disciplina de Gastroenterologia, São Paulo, SP, Brasil.

**Keywords:** Sarcopenia, neoplasms, nutrition assessment, Sarcopenia, neoplasias, avaliação nutricional

## Abstract

**Background:**

Cancer is a malignant disease characterized by the accumulation of genetic alterations with uncontrolled cell growth. In 2022, approximately 20 million new cancer cases and nearly 10 million deaths occurred globally. Cancer patients are at high risk for malnutrition due to the disease and treatment. Sarcopenia can negatively affect clinical outcomes and is associated with a higher likelihood of adverse results, including falls, fractures, low physical performance, and mortality.

**Objective:**

The aim of this study was to determine whether ultrasound of the gastrocnemius muscle can be a method for detecting the risk or presence of sarcopenia in patients with digestive system cancer.

**Methods:**

This is a cross-sectional study conducted with patients diagnosed with digestive system cancer, aged 18 years or older, undergoing treatment. The 2018 EWGSOP algorithm was used to evaluate sarcopenia, and participants were classified into two groups: one group at risk for sarcopenia or sarcopenic and another group as non-sarcopenic. A portable ultrasound (BodyMetrix™ BX2000 ultrasound) was used to measure the thickness of the gastrocnemius muscle.

**Results:**

The diagnostic performance of ultrasound (US) and calf circumference (CC) in detecting sarcopenia was evaluated by the area under the ROC curve. The area under the curve for US was 0.940, with 80% sensitivity and 100% specificity, and for CC, it was 0.889, with 72% sensitivity and 95% specificity. We found a negative correlation between the measure of the gastrocnemius muscle by ultrasound (US) and Sarc-CalF, and a positive correlation between the US and calf circumference (CC).

**Conclusion:**

The evaluated method shows potential for clinical application, suggesting it could be a fast and sensitive clinical tool. However, further studies are needed to establish robust evidence base and achieve a more accurate sarcopenia diagnosis for early multidisciplinary intervention.

## Abstract (key points)

•Cancer patients with digestive system malignancies are at high risk of malnutrition and sarcopenia, which adversely impact clinical outcomes and survival.

• Gastrocnemius muscle thickness assessed by portable ultrasound demonstrated excellent diagnostic accuracy for identifying sarcopenia risk.

• Ultrasound showed superior performance (AUC 0.940; 80% sensitivity, 100% specificity) compared with calf circumference.

• Gastrocnemius ultrasound measurements correlated positively with calf circumference and negatively with Sarc-CalF scores.

• Muscle ultrasound may represent a rapid, noninvasive, and clinically feasible tool for early sarcopenia screening in oncology settings.

## RESUMO

**Contexto:**

O câncer é uma doença maligna caracterizada pelo acúmulo de alterações genéticas com crescimento celular descontrolado. Em 2022, ocorreram aproximadamente 20 milhões de novos casos de câncer e cerca de 10 milhões de mortes em todo o mundo. Pacientes com câncer apresentam alto risco de desnutrição devido à própria doença e ao tratamento. A sarcopenia pode impactar negativamente os desfechos clínicos e está associada a uma maior probabilidade de resultados adversos, incluindo quedas, fraturas, baixo desempenho físico e mortalidade.

**Objetivo:**

O objetivo deste estudo foi determinar se o exame de ultrassonografia do músculo gastrocnêmio pode ser um método para detectar o risco ou a presença de sarcopenia em pacientes com câncer do sistema digestivo.

**Métodos:**

Trata-se de um estudo transversal realizado com pacientes diagnosticados com câncer do sistema digestivo, com 18 anos ou mais, em tratamento. Foi utilizado o algoritmo da EWGSOP de 2018 para avaliar a sarcopenia, e os participantes foram classificados em dois grupos: um grupo em risco de sarcopenia ou sarcopênico e outro grupo como não sarcopênico. Utilizou-se um aparelho portátil de ultrassom (ultrassom BodyMetrix™ BX2000) para medir a espessura do músculo gastrocnêmio.

**Resultados:**

O desempenho diagnóstico da ultrassonografia (US) e da circunferência da panturrilha (CC) na detecção de sarcopenia foi avaliado pela área sob a curva ROC. A área sob a curva para a US foi de 0,940, com 80% de sensibilidade e 100% de especificidade; para a CC, foi de 0,889, com 72% de sensibilidade e 95% de especificidade. Observou-se uma correlação negativa entre a medida do músculo gastrocnêmio por ultrassom e o Sarc-CalF, e uma correlação positiva entre a US e a circunferência da panturrilha.

**Conclusão:**

O método avaliado demonstra potencial para aplicação clínica, sugerindo ser uma ferramenta clínica rápida e sensível. No entanto, mais estudos são necessários para estabelecer uma base de evidências robusta e alcançar um diagnóstico de sarcopenia mais preciso, possibilitando intervenção multidisciplinar precoce.

## INTRODUCTION

Cancer is a malignant disease characterized by the accumulation of genetic alterations with uncontrolled cell growth, with potential for invasion of adjacent tissues or distant organs. In 2022, approximately 20 million new cancer cases and nearly 10 million deaths occurred globally. The global estimate for 2050 is 35 million cases, a 77% increase compared to 2022^1^ ^-^ ^3^.

Cancer patients are at high risk for malnutrition due to the disease and treatment. The severity of malnutrition can be influenced by factors such as age, stage, tumor location, and type. The inflammatory response causes anorexia, which may result in significant weight loss, changes in body composition, and decline in physical function. In addition to anorexia, vomiting and diarrhea can occur, affecting nutritional status, leading to a decrease in lean mass and the onset of sarcopenia^4^ ^-^ ^7^.

Sarcopenia can negatively affect clinical outcomes and is associated with a higher likelihood of adverse results, including falls, fractures, low physical performance, and mortality. The 2018 “European Working Group on Sarcopenia in Older People” (EWGSOP2) questionnaire provided a diagnostic algorithm for sarcopenia that begins with screening using the SARC-F, followed by strength assessments such as the Handgrip Strength Test (HGS), evaluation of muscle volume and body composition through analysis of appendicular skeletal muscle mass by dual-energy X-ray absorptiometry (DEXA), total body skeletal muscle mass analyzed by bioimpedance (BIA), and the cross-sectional area of the muscle at mid-thigh by computed tomography or magnetic resonance imaging^8^.

Although the methods for evaluating body composition are highly accurate, not all are feasible for use in clinical practice. In this context, ultrasound has been studied as a low-cost method that can be easily implemented and can identify short-term changes in muscle structure. However, standardized validated protocols are still lacking, as studies vary significantly in patient positioning, muscles evaluated, anatomical landmarks, analyzed parameters, and techniques used^9^ ^,^ ^10^.

Given the clinical condition of cancer patients and the importance of early identification of nutritional status for early and effective therapeutic approaches, studies involving tools to assess body composition, along with other methods such as questionnaires, function tests, performance assessments, and evaluations of emotional and social status, are necessary for accurate diagnosis and more targeted intervention.

The aim of this study was to determine whether ultrasound of the gastrocnemius muscle can be a method for detecting the risk or presence of sarcopenia in patients with digestive system cancer.

## METHODS

This is a cross-sectional study conducted with patients diagnosed with digestive system cancer, aged 18 years or older, undergoing treatment at the Gastrointestinal Oncology Clinics of the HSP-UNIFESP/EPM complex. Inclusion criteria were patients with digestive system cancer receiving oncological treatment or clinical follow-up. Patients with edema, lower limb amputation, inability to lie in a supine position or walk, pacemaker use, or limitations that prevented them from answering questions or performing measurements were excluded.

The variables collected were name, age, gender, ethnicity, profession, education level, diagnosis, stage, therapy used, and “ECOG Performance Status” (PS-ECOG)^11^.

Weight and height were measured using the Welmy W 200® scale and Sanny® stadiometer, respectively. Body mass index (BMI) was calculated, and the classification was based on the World Health Organization’s guidelines for individuals aged 20 to 59 years, while for elderly individuals (>65 years), the cutoff points proposed by Lipschitz were used^12^ ^,^ ^13^.

For all participants, the Sarc-F questionnaire was administered, with the additional measurement of calf circumference (CC) to perform the Sarc-CalF^8^ ^,^ ^14^ ^,^ ^15^.

The 2018 EWGSOP algorithm was used to evaluate sarcopenia, and participants were classified into two groups: one group at risk for sarcopenia (patients with SARC Calf >10 and/or reduced muscle mass and/or decreased muscle strength) or sarcopenic, and another group as non-sarcopenic. Sarcopenia was assessed by handgrip strength using the Jamar® digital dynamometer (decreased handgrip strength <27 kg for men or <16 kg for women). To confirm sarcopenia by detection low muscle mass, bioimpedance analysis was used (Bodystat® and Quadscan 4000 model, with four frequencies: 5, 50, 100, and 200 kHz). The Time Up and Go (TUG) test measured physical performance severity by timing how long it took to stand up, walk 3 meters^16^.

A portable ultrasound (BodyMetrix™ BX2000 ultrasound) was used to measure the thickness of the right gastrocnemius muscle. The measurement was taken in the most prominent area of the calf, at the medial gastrocnemius, with the foot flat on the ground and the leg relaxed, maintaining a 90-degree angle.

The circumference of the calf was measured at its largest point and was used to determine muscle position. From this measurement, one-third of half the circumference was calculated to identify the measurement site. A mark was placed on the posteromedial aspect of the leg and used as the reference point for positioning the transducer during the ultrasonographic evaluation, aiming to standardize and ensure the reproducibility of the measurements among participants. For the examination, the device was connected to a computer running the BodyViewProFit® software, and a conductive gel was applied to the ultrasound probe. The evaluation was performed from top to bottom at the marked point. All measurements and images were obtained by the researcher after training conducted by the company Bodymetrix®.

### Statistical analysis

The exploratory data analysis included descriptive statistics such as mean, standard deviation, percentiles, minimum and maximum values for numerical variables, and frequency and proportion for categorical variables. To assess the behavior of continuous variables, descriptive statistics, histogram and boxplot graphs, and the Shapiro-Wilk test for the theoretical normality assumption were used. For comparative analysis of numerical variables between the two groups based on sarcopenia, the Mann-Whitney test was applied. The correlation analysis between two variables (ultrasound of the gastrocnemius muscle and BIA parameters) was assessed using the Spearman correlation coefficient, defined as strong |0.50 - 1.00|, moderate |0.30 - 0.49|, and weak |0.10 - 0.29|^17^ ^-^ ^19^.

The diagnostic performance of the methods for detecting sarcopenia was evaluated using the area under the ROC curve (AUC). The optimal cutoff points for predicting the outcome were identified using Youden’s method. Sensitivity, specificity, predictive values, and likelihood ratios for these cutoff points were also calculated. To compare the AUCs for gastrocnemius muscle ultrasound and calf circumference, the DeLong test was used. *P*-values <0.05 were considered significant. All analyses were conducted using version 4.3.2 of the R programming language^20^.

## RESULTS

The sample consisted of 45 participants with a mean age of 64 (±10.6) years. The majority were male (55.56%), had completed elementary school (65.91%), and were white (47.73%). All patients had adenocarcinoma, with colorectal cancer (CRC) the most common, affecting about 44% of the sample. Most patients (68.18%) were classified as ECOG 0-1. Eighteen (40%) patients had advanced disease (stage IV). Most patients underwent chemotherapy (Table 1). Table 2 presents the anthropometric measures used to assess participant’s nutritional status**.** The mean calf circumference (CC) was 34.0±4.0 cm, with 22.2% of women and 28.9% of men having values below the sex-specific cutoff points. The mean body mass index (BMI) was 22.5±4.6 kg/m², with a predominance of individuals classified as normal weight (44.4%) and 40.0% classified as underweight. Nearly half of the participants (46.7%) had reduced muscle mass, as assessed by bioelectrical impedance analysis (BIA). The mean SARC-CalF score was 7.5±6.4, with 37.8% of individuals at increased risk of sarcopenia. Lower gastrocnemius muscle thickness values were observed in patients (Table 2) at risk for sarcopenia and those with sarcopenia compared to the non-sarcopenic group (*P*<0.05).

**TABLE 1:** Characteristics of the patients studied.

Age (mean ± SD)		64.2	±10.6
		N	%
Gender	Male	25	55.6
	Female	20	44.4
Site of the tumor	Gastric	14	31.1
	Biliopancreatic	7	15.6
	Colorectal	20	44.4
Clinical Stage	I-II	16	35.6
	III	11	24.4
	IV	18	40.0
Treatment	On chemotherapy	40	88.9
	Others	5	11.1

**TABLE 2:** Anthropometric measures used to assess nutritional state.

		mean±SD	N (%)
CC (cm)		34.0±4.0	
Low CC	Women (≤33 cm)		10 (22.2)
	Men (≤34 cm)		13 (28.9)
BMI (kg/m²)		22.5±4.6	
Classification	Underweight		18 (40.0)
	Normal weight		20 (44.4)
	Overweight-obese		7 (15.6)
**BIA**		42.1±11.7	
Low BIA (< )			21 (46.7)
Sarc-CalF		7.5±6.4	
Risk of sarcopenia	11-20		17 (37.8)

SD: standard deviation. CC: calf circumference. BMI: body mass index (kg/m²). BIA: bioelectrical impedance analysis.

**FIGURE 1:**
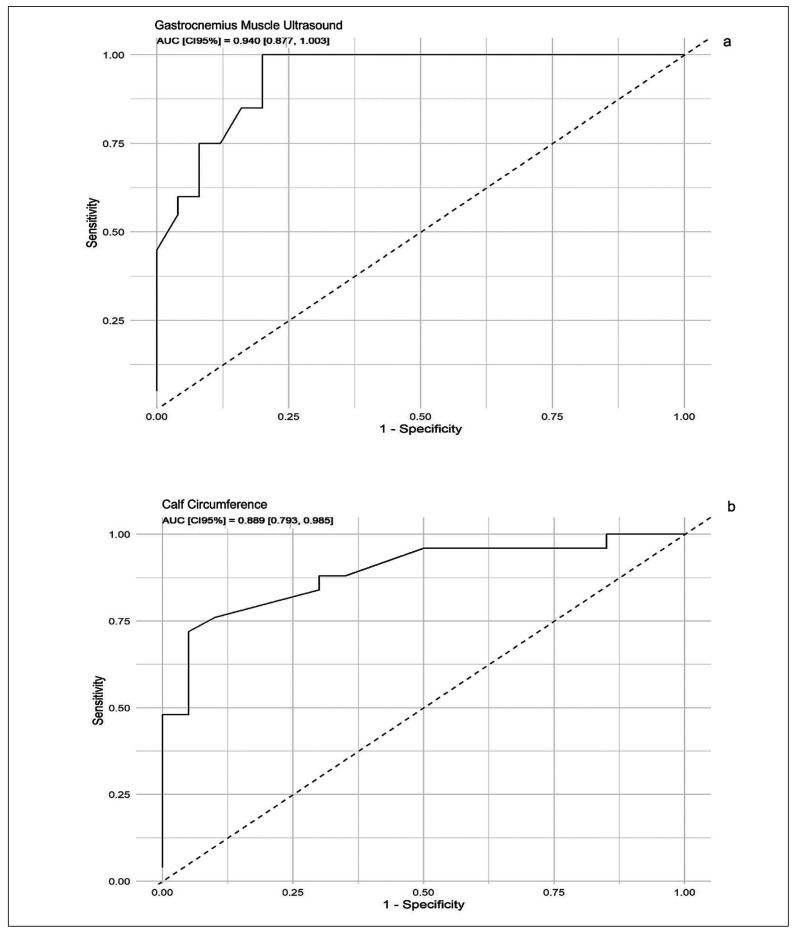
ROC curve for the variable gastrocnemius muscle ultrasound (A) and calf circumference (B).

**FIGURE 2:**
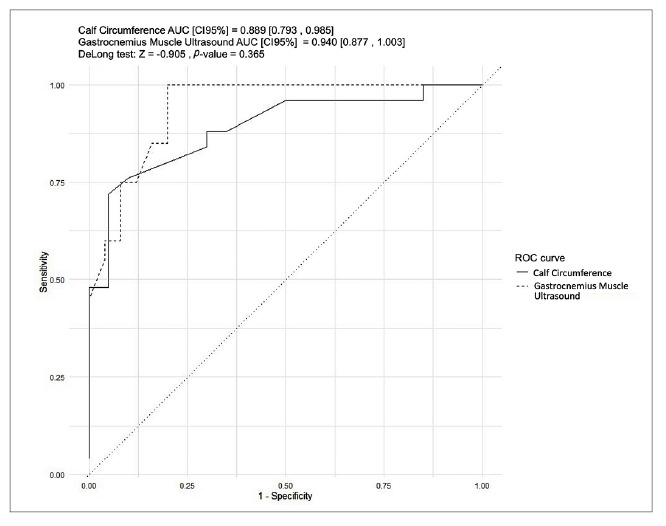
Comparison between the differences in calf circumference (CC) and gastrocnemius ultrasound (US).

The diagnostic performance of ultrasound (US) and calf circumference (CC) in detecting sarcopenia was evaluated by the area under the ROC curve. As shown in Figure 1, the area under the curve for US (a) was 0.940, with 80% sensitivity and 100% specificity, and for CC (b), it was 0.889, with 72% sensitivity and 95% specificity. Using the Youden method (Table 3), the cutoff point for the gastrocnemius muscle was 11.5 mm. No significant difference was observed between the gastrocnemius ultrasound and calf circumference (DeLong test) (Figure 2).

**TABLE 3:** AUC, Cut off, sensitivity and specificity of ultrasound of the gastrocnêmio muscle and calf circumference.

	Cut off	AUC	CI	Sensitivity	Specificity	*P*
USG	11.5	0.94	0.87-1.00	0.80	1.00	0.365
CC	33	0.889	0.79-0.98	0.72	0.95	

AUC: area under the curve. CI: confidence interval. USG: ultrasound of the gastrocnemio. CC: calf circumference.

We found a negative correlation between the ultrasound (US) measure of the gastrocnemius muscle by and Sarc-CalF, and a positive correlation between the US and calf circumference (CC). There was also a significant correlation between the US and the BIA parameters (Table 4).

**TABLE 4:** Spearman coefficient correlation between the ultrasound of the gastrocnemio and SARC Calf, CC, MM and MMLG.

Ultrassom of the gastrocnêmio muscle versus	r	CI95%	R2	*P*
SARC CalF	-0.55	-0.73-0.31	0.30	<0.001
CC	0.71	0.53-0.83	0.50	<0.001
Lean mass	0.51	0.24-0.71	0.26	<0.001
Fat free mass	0.66	0.42-0.81	0.43	<0.001

CC: calf circumference. r: spearman coefficient risk. CI: confidence interval.

## DISCUSSION

In this study, it was observed that the gastrocnemius muscle thickness in patients with digestive system cancer can predict the risk or presence of sarcopenia. To date, this is the first study to measure gastrocnemius muscle thickness was measured using ultrasound (US) to assess sarcopenia in patients with digestive system cancer.

Fu et al.^21^ conducted a systematic review to assess the diagnostic accuracy of ultrasound for identifying sarcopenia. The gastrocnemius, rectus femoris, and femoral quadriceps muscles were the most frequently assessed muscle groups because they are easier to measure and are associated with mobility and activities of daily living. Muscle thickness was the most commonly used measure, followed by the cross-sectional area. Eight studies were considered eligible for evaluating the diagnostic value of gastrocnemius muscle thickness for sarcopenia, and the meta-analysis showed moderate diagnostic accuracy, with a sensitivity of 82% (95%CI 71-90%) and specificity of 64% (95%CI 48-77%).

Alvarez et al.^22^ correlated the cross-sectional thickness of the medial gastrocnemius with appendicular lean mass estimated by DEXA and calf circumference, with a sensitivity and specificity of 77.8%. A significant positive correlation was also demonstrated with calf circumference (r=0.651, *P*<0.001).

When evaluating which muscles (head, neck, upper limbs, and lower limbs) showed detectable thickness changes through ultrasound in sarcopenic patients, Barotsis et al.^23^ concluded that neck and lower limb muscle thickness could be used to predict sarcopenia with high sensitivity and specificity. The likelihood of sarcopenia was 11.9 times higher for low rectus femoris thickness and 11.3 times higher for low medial gastrocnemius thickness. The hypothesis for the lack of significant changes in the masseter, tibialis anterior, and arm muscles in sarcopenic individuals was that these muscles are more prone to fat infiltration.

Our study reported sensitivity and specificity comparable to those in the other studies^25^ ^,^ ^22^ ^,^ ^23^, indicating that gastrocnemius muscle ultrasound (US) can be a promising and easily applicable method in clinical practice. However, given the limited number of studies in the literature, further research is needed to assess the accuracy of this tool using similar methodologies and specific populations.

Kuyumcu et al.^24^ evaluated the muscle and non-muscle compartments of the calf in 100 hospitalized elderly patients using ultrasound and found that the gastrocnemius thickness was lower in those with sarcopenia. They suggested that calf circumference should not be assessed independently, as non-muscular compartments (such as adipose tissue or edema in the subcutaneous fat layer) may interfere with the measurement.

Aycicek et al.^25^ studied 136 Turkish patients admitted to a geriatrics outpatient clinic to compare the lean mass measured by BIA with gastrocnemius muscle thickness measured by ultrasound and determined the cutoff values. To predict sarcopenia, the best cutoff point for gastrocnemius muscle thickness was ≤12.3 mm in women and men (87.5% sensitivity and 83.75% specificity for women; 83.33% sensitivity and 87.18% specificity for men). Another study, conducted with 195 elderly Japanese, defined the gastrocnemius muscle ultrasound cutoff point as <11.6 mm (83% sensitivity, 73% specificity)^26^. The cutoff points described for the Turkish and Japanese populations were similar to those observed in our study. To improve the accuracy of ultrasound for detecting sarcopenia, multicenter studies with a larger number of participants are necessary to confirm its effectiveness.

According to the present study, there is an inversely proportional relationship between gastrocnemius muscle thickness measured by ultrasound and the SARC-CalF score. This finding strengthens the hypothesis that gastrocnemius muscle thickness, measured by ultrasound, can predict the risk of sarcopenia, as higher SARC-CalF scores are associated with a higher risk of sarcopenia. We believe this is the first study to correlate the Sarc-CalF instrument with gastrocnemius muscle ultrasound. Fu et al.^27^ evaluated the diagnostic value of Sarc-F and Sarc-CalF for sarcopenia screening in 309 cancer patients. Sarcopenia was determined through low muscle mass from tomography and handgrip strength. The prevalence of low muscle mass from tomography and sarcopenia was 85.1% and 50.5% according to Western criteria. In this study, Sarc-CalF significantly increased the sensitivity and overall diagnostic accuracy of Sarc-F in cancer patients.

In this study, a significant correlation was found between gastrocnemius muscle thickness measured by ultrasound and lean mass parameters. A systematic review that evaluated 24 studies involving 3,607 oncology patients concluded that BIA is an accurate method for detecting sarcopenia and a viable alternative to tomography, dual-energy X-ray absorptiometry (DEXA), and magnetic resonance imaging (MRI) in clinical practice^28^.

Study limitations include the small number of patients included and the lack of comparison between ultrasound and gold-standard methods for noninvasive body composition evaluation, such as MRI and CT scans.

Despite the mentioned limitations, we observed that gastrocnemius muscle thickness measured by ultrasound can predict the risk or presence of sarcopenia in patients with digestive system cancer. The method demonstrated high sensitivity and specificity in identifying patients with the risk, presence, or absence of sarcopenia. Calf circumference also identified and differentiated patients with and without sarcopenia; however, its performance was inferior to that of gastrocnemius muscle ultrasound.

The evaluated method shows potential for clinical application, suggesting it could be a fast and sensitive clinical tool. However, further studies are needed to establish robust evidence base and achieve a more accurate sarcopenia diagnosis for early multidisciplinary intervention.

## Data Availability

The research data are presented within the article itself (available in Tables 1-4 and Figures 1 and 2).
